# Pictogram comprehension and medication-use literacy among undergraduate students: a cross-sectional survey study

**DOI:** 10.1080/20523211.2025.2522312

**Published:** 2025-06-27

**Authors:** Kritsanee Saramunee, Bunleu Sungthong, Chatmanee Taengthonglang, Wiraphol Phimarn

**Affiliations:** a Social Pharmacy Research Unit, Faculty of Pharmacy, Mahasarakham University, Kantharawichai, Thailand; b Integrative Pharmaceuticals and Innovation of Pharmaceutical Technology Research Unit, Faculty of Pharmacy, Mahasarakham University, Mahasarakham, Thailand; c Pharmacy Department, Surin Hospital, Surin, Thailand

**Keywords:** Health literacy, pictograms, drug labels, university student

## Abstract

**Background:**

Pictograms are widely used in pharmacy practice to enhance patient understanding, especially in contexts where language or health literacy barriers exist. However, limited data are available on the comprehension of United States Pharmacopeia Convention: Drug Information for the Health Care Professional (USP-DI) pictograms among Thai undergraduate students. This study assessed the understanding of USP-DI pictograms and medication-use literacy, and explored factors associated with pictogram comprehension.

**Methods:**

A cross-sectional survey using a structured questionnaire was conducted among 637 university students between August 2023 and April 2024. The Medication Use Literacy Test (MULT) assessed literacy, and the USP-DI pictogram comprehension test evaluated visual understanding. Binary logistic regression was used to identify factors associated with comprehension levels.

**Results:**

Among the 637 participants, the MULT revealed high literacy, with over 90% of questions answered correctly. The mean score for USP-DI pictogram comprehension was 21.82 ± 3.82. Students in health sciences programmes demonstrated the highest proficiency in both tests, followed by those in social sciences and science and technology programmes, with statistically significant differences (*P* < 0.05). Logistic regression analysis revealed that students aged >20 years had lower pictogram comprehension than younger students (odds ratio [OR] = 0.47; 95% confidence interval [CI]: 0.25–0.88; *P* = 0.02). Students with a Grade Point Average <3.00/4.00 (OR = 0.55; 95% CI: 0.32–0.96; *P* = 0.04) and those from non-health science faculties (OR = 0.04; 95% CI: 0.02–0.08; *P* < 0.001) also showed significantly lower comprehension levels.

**Conclusions:**

This study highlights disparities in pictogram comprehension among Thai undergraduates and affirms the influence of academic discipline, age, and academic performance. The findings support the need for targeted educational strategies to enhance medication-use literacy and pictogram understanding.

## Introduction

Medicine is one of the four critical factors influencing human well-being (Puipuroy et al., 2020; Vargas-Peláez et al., 2017). Health literacy (HL), defined as the ability to access, understand, and use health information to make informed decisions, is a key determinant of health outcomes worldwide (Nutbeam, 2008). A central component of HL is medication literacy, which directly affects how individuals interpret drug labels, adhere to therapy, and prevent medication-related harm (Pouliot et al., 2018). Despite advances in patient education, studies consistently show that a significant proportion of individuals – especially those with limited literacy or non-native language proficiency struggle to understand medication instructions, often resulting in misuse and suboptimal treatment outcomes (Bekker et al., 2020; Jeetu & Girish, 2010).

To address this issue, the use of visual communication tools, such as pictograms, has gained increasing attention. Pictograms are standardised images that convey essential instructions or precautions associated with medication use, helping bridge communication gaps and reduce reliance on text-based information (Dowse & Ehlers, 2005). Their effectiveness is supported by the picture superiority effect, which suggests that images are more easily processed, retained, and recalled than verbal information alone (Baadte & Meinhardt-Injac, 2019). Empirical evidence indicates that patients prefer pictorial labels and that these tools significantly improve comprehension, especially among individuals with low literacy levels (Katz et al., 2006).

An effective approach to overcome these obstacles in communication is the utilisation of pictograms, which are visually appealing two-dimensional images designed to capture attention and transmit information without the need for language or words (Dowse, 2023). The pictogram literature presents compelling proof that such images can enhance understanding and awareness of health and medicine-related information (Mohammad et al., 2022). Pictograms on prescription labels have a favourable impact on health outcomes in terms of adherence and self-efficacy (Merks et al., 2021; Sletvold et al., 2020; Yin et al., 2008). Pictograms are highly effective communication aids for pharmacists in counselling patients regarding medicine usage, especially in situations when communication obstacles exist (Browne et al., 2019; Mbanda et al., 2021).

The United States Pharmacopeia Convention: Drug Information for the Health Care Professional (USP-DI) has established a system of pictograms to facilitate communication between patients and healthcare providers. This system includes 81 universally descriptive symbols that must not be altered or combined with other images. These pictograms are available for free and can be accessed by the public on the USP website (https://www.usp.org/download-pictograms). Given the potential for varied interpretations, the meaning of each image must be communicated explicitly to the patients. Moreover, the images employed should align with the beliefs, society, and culture of the target audience to accurately convey the intended meaning (Lin et al., 2021).

Low HL is more prevalent among those with limited educational backgrounds (Shah et al., 2022). In a study on 78 female ambulatory patients, Ngoh and Shepherd discovered that the use of visual aids significantly enhanced the patients’ understanding of their medication regimens (Ngoh & Shepherd, 1997). The incorporation of pharmaceutical pictograms was particularly effective in improving patients’ comprehension of instructions and promoting adherence to prescribed treatments (Houts et al., 2006; Mansoor & Dowse, 2003).

A previous study evaluated the interpretive accuracy of 23 USP-DI pictograms and of the ones specifically developed for use in South Africa. The findings revealed that participants had a better understanding of the South African pictogram than the USP-DI pictogram. Notably, at least 85% of the responses met the American National Standards Institute (ANSI) criteria for understanding pictograms, demonstrating cognitive recognition of the pictograms (Dowse & Ehlers, 2001). However, Vaillancourt et al. conducted a study to validate the comprehension of nine pictograms designed to enhance medication safety and assessed the long-term recall of these pictograms among pharmacy students. This study involved 101 pharmacy students in phase 1 and 67 pharmacy students in phase 2. Initially, four pictograms met the 67% threshold for comprehension, and seven of the nine pictograms were validated (Vaillancourt et al., 2018). A South African study examined the relationship between HL and comprehension of pictograms indicating drug use and side effects among a population with low literacy and limited English proficiency. The comprehension of 10 locally developed pictograms was evaluated, yielding a mean score of 7.9 out of 10. Eight out of ten pictograms met the International Organization for Standardization criterion of 66.7% correct comprehension. Only 15.6% of the participants demonstrated adequate HL. A significant correlation between HL and pictogram comprehension was established (*P* = 0.002). Higher rates of pictogram misinterpretation were observed in participants with low HL, whereas adequate HL was associated with superior comprehension. Pictogram comprehension was negatively associated with age (*P* < 0.006) and positively associated with education (*P* < 0.001) and English proficiency (*P* < 0.001) (Dowse et al., 2023).

Effective medication use often depends on patients’ understanding of medication instructions; however, this process is frequently hindered by limited HL and language barriers, particularly in low-resource settings (Katz et al., 2006). Pictograms – visual symbols that convey medication-related information – have emerged as important tools to bridge these communication gaps. Several international studies have examined pictogram comprehension in diverse populations, including pharmacy students. For example, a study by Yasmin et al. in Pakistan found high recognition rates (98%) for USP-DI and locally developed pictograms among pharmacy and non-pharmacy students, with a preference for USP-DI images (Yasmin et al., 2014). Similarly, in South Africa, pharmacy students participating in a rural outreach programme reported that over 95% believed pictograms enhanced communication with low-literacy patients (Dowse, 2023). However, these studies were conducted within specific cultural and linguistic contexts and primarily involved students with formal pharmacy training.

Despite the growing global adoption of pictogram-based labelling, limited evidence exists regarding comprehension among university students outside pharmacy programmes, particularly in non-Western settings. In Thailand – where multigenerational households are common and students often participate in caregiving roles – pictogram comprehension may be vital to ensuring safe medication use.

Yet, no published studies have assessed understanding of standardised pictograms, such as those from the USP-DI, among Thai undergraduates across health science and non-health science disciplines. Given the increasing role of students as informal medication advisors in Thai households, this gap warrants attention. The present study aims to assess the comprehension of USP-DI pictograms among Thai undergraduate students and to examine differences between health science and non-health science faculties. Findings may inform future health education strategies and contribute to the development of more effective pictogram-based interventions in Thailand. This study was aimed at (1) exploring the understanding of USP-DI pictograms and assessing medication-use literacy among undergraduate students of a university in Thailand, (2) investigating the comprehension of USP-DI pictograms among these students, and (3) examining the relationship between various factors and the level of comprehension of pictograms.

## Methods

This cross-sectional survey was conducted to assess the comprehension of USP-DI pictograms among undergraduate students at a university in Thailand. Ethical approval was obtained from the university’s research ethics committee (Ref: 010/2557). Informed consent was obtained from all the participants, and all survey responses were anonymized prior to analysis.

### Study population

The study population comprised undergraduate students enrolled at the university during the 2023–2024 academic year who consented to participate.

### Sample size

The required sample size was calculated using the Taro Yamane formula (Yamane, 1973), targeting a minimum of 514 participants aged 18 years or older. Data collection was conducted between August 2023 and April 2024.

### Data collection instruments

#### Demographic and health variables

The survey instrument included eight demographic and health-related variables: gender, age, faculty, education level, underlying disease, number of family members, monthly income, and frequency of hospital visits or medication use over the previous year. The frequency of hospital visits or medication use was categorised as follows: less than once per month, once per month, twice per month, three times per month, and more than three times per month.

#### Medication Use Literacy Test (MULT)

The Thai version of the MULT was used to evaluate functional literacy related to medication use. The test consists of true-or-false questions based on typical medication instructions, requiring respondents to assess the appropriateness of specific actions. A score of ≥9 indicated adequate literacy, whereas a score of ≤8 denoted low literacy. The MULT was chosen for its focus on practical medication-related tasks, such as reading, interpreting, and applying drug label information. It has been previously validated in Thai student and patient populations (Chaijinda et al., 2007).

#### USP-DI pictogram comprehension test

The USP-DI pictogram comprehension test consisted of 30 pictograms. A subset relevant to medications commonly used in Thai clinical settings was selected in consultation with clinical pharmacists and hospital practitioners. These pictograms represented external preparations, administration devices (e.g. inhalers, injectables), safety warnings (e.g. drowsiness, pregnancy, lactation), and special instructions. Participants were asked to provide brief written descriptions of each programme’s meaning. Open-ended responses were used to evaluate true comprehension, rather than mere recognition.

#### Questionnaire validation

The questionnaire underwent iterative design and content validation by the research team. Reviewers included three clinical pharmacy lecturers, three hospital pharmacists, and four community pharmacists. Reliability testing was conducted with 15 volunteers. Based on this pilot phase, minor modifications were made to improve clarity and reliability. Internal consistency was confirmed with Cronbach’s alpha coefficients of 0.71 for the USP-DI pictogram comprehension test and 0.88 for the MULT, indicating acceptable reliability.

#### Outcome measures

The primary outcome measure was the USP-DI pictogram comprehension test. Following completion of the questionnaire by the participants, the investigator assessed the accuracy of the responses by comparing them with the correct interpretation of each pictogram. Each response was scored based on whether it aligned with the image label: 1 point was awarded for correct interpretation, and 0 points were assigned for incorrect or inaccurate interpretation. Similarly, the MULT comprised true-or-false questions. The participants self-administered the questionnaire and marked their responses directly. Correct responses earned 1 point, whereas incorrect responses received 0 points.

#### Survey procedure

Survey forms were distributed to undergraduate students using a convenience-based street survey approach designed to enhance diversity and approximate representativeness of the student population. Data collection took place across multiple faculty buildings and common university areas (e.g. canteens, libraries, student union zones). Researchers varied the times and days of data collection, including mornings, afternoons, and early evenings on weekdays and weekends, to capture students from different faculties and schedules. At each location, students were approached opportunistically and invited to participate, ensuring variation in academic backgrounds and minimising time-based selection bias. The researcher provided detailed explanations of the data collection process and the purpose of the research. The participants completed the questionnaire independently, which took approximately 10–20 min. Upon completion, the responses were collected immediately. Each completed form was reviewed for accuracy and completeness. If any responses were incomplete or incorrectly completed, participants were asked to correct their answers immediately to ensure the integrity of the data for subsequent analysis.

### Data analysis and statistics

The researcher utilised the collected data and employed the STATA version 15 statistical software for data verification and analysis. The following statistical methods were applied: Descriptive statistics were used to characterise the study population based on variables such as age, gender, academic year, and Grade Point Average (GPA), presented in terms of frequency, percentage, mean, and standard deviation. The range from minimum to maximum values was also reported.

To assess the understanding of the USP-DI pictogram, responses were evaluated using open-ended questions regarding the number of pills taken per dose, frequency of administration, and timing relative to meals, scored as correct or incorrect, and presented as percentages.

For multivariate analysis, binary logistic regression was employed. The analyzed variables included gender (male vs. female), age (<20 years vs. ≥ 20 years), frequency of hospitalisations (<1 time per month vs. ≥ 1 time per month), cumulative GPA (<3.00 vs. 3.00–4.00), and faculty affiliation (health sciences vs. non-health sciences).

According to predefined comprehension criteria, participants were considered proficient in USP-DI pictogram interpretation if they scored at least 26 out of 30 points (≥85%), in accordance with the ANSI Z535.3 standard for acceptable pictogram comprehension (ANSI, 2011). For the MULT, scores of 0–8 were classified as indicating inadequate medication-use literacy, whereas scores of 9–10 indicated adequate literacy, based on the validated classification used in previous Thai studies (Chaijinda et al., 2007).

All variables were transformed into dummy variables, with male sex, age <20 years, < 1 hospitalisation per month, cumulative GPA <3.00, and non-health sciences faculty coded as 0. A USP-DI pictogram comprehension score below 26 points and a MULT score of 0–8 points were used as criteria for analysis. Variables with a univariate *P*-value <0.2 were selected for inclusion in the binary logistic regression model. Odds ratios (ORs) and 95% confidence intervals (CIs) were calculated to determine the significance of the associations, with the significance level set at *P* < 0.05.

## Results

### General characteristics of the sample

A total of 637 participants were included in the study, predominantly females, comprising 72.1% of the sample, and males accounting for 27.9%. Participants were drawn from various faculties of the selected university, with 55.7% aged younger than 20 years and 44.3% aged 20 years or older. The Faculty of Accounting and Management had the highest number of respondents, totalling 140 (22.0%), whereas the Faculty of Cultural Sciences had the lowest, with only 2 (0.3%) respondents. In terms of academic performance, the highest GPA range among the sample was 3.00–3.49, representing 21.2% of the total. Most participants (90.4%) reported no underlying diseases. Regarding hospital visits, 52.6% reported visiting less than once per month. The most common family size reported was four members, accounting for 52.4% of the total. On average, the students reported a monthly income of 5001–10,000 baht, representing 25.7% of the sample (Table 1).

**Table 1.** General information of the sample.

General information	*N* = 637	Percent
Gender		
Female	459	72.1
Age (years)		
≤20	355	55.7
Average age	20.57 ± 1.89	
Year		
1	241	37.8
2	93	14.6
3	123	19.3
4	75	11.8
5	31	4.9
6	74	11.6
Faculty		
Education	26	4.1
Humanities and Social sciences	81	12.7
Fine arts	8	1.3
Tourism and Hospitality	27	4.2
College of Politics and Administration	35	5.5
College of Music	7	1.1
Cultural studies	2	0.3
Law	37	5.8
Science	31	4.9
Technology	26	4.1
Information science	29	4.6
Architecture, urban planning and creative arts	15	2.4
Engineering	17	2.7
Environment and Resource studies	18	2.8
Nursing	10	1.6
Pharmacy	113	17.7
Public health	7	1.1
Medicine	4	0.6
Veterinary medicine	4	0.6
Accounting and Management	140	22.0
GPAX		
≤1.99	7	1.1
2.00–2.49	67	10.5
2.50–2.99	102	16.0
3.00–3.49	135	21.2
3.50–4.00	62	9.7
Underlying disease		
No	576	90.4
Allergic rhinitis	25	3.9
Arrhythmia	1	0.2
Asthma	11	1.7
Hyperthyroidism	6	0.9
Dyspepsia	7	1.1
Migraine	5	0.8
Systemic lupus erythematosus (SLE)	1	0.2
Cardiovascular disease	1	0.2
Anemia	2	0.4
Thalassemia	1	0.2
Hepatitis B virus (HBV) infection	1	0.2
Number of family members		
1	7	1.1
2	16	2.5
3	80	12.6
4	334	52.4
5	123	19.3
6	51	8.0
7	21	3.3
8	5	0.8
Number of hospitals admitted in the past 1 year (time/month)		
<1	335	52.6
1	74	11.6
2	23	3.6
3	42	6.6
>3	163	25.6
Monthly income (baht)		
≤3000	11	1.7
3001–5000	110	17.3
5001–10,000	164	25.7
>10,000	23	3.6
Average monthly income	7367.94 ± 5.44	

### MULT score

In the sample of 637 participants, the evaluation of the MULT revealed a high level of accuracy across all questions, with over 90% correct responses for each item. In particular, respondents demonstrated exceptional understanding of item 7, where 98.4% correctly identified that ‘Mr. Dang took the medicine without reading the label that suggested how to take the medicine.’ Similarly, item 8, which assessed the understanding of medication timing relative to meals (‘Mr. Som received the medicine before meals. When Mr. Som finished taking the medicine, he immediately resumed eating.’), also received a high correct response rate (Table 2).

**Table 2.** MULT score of participants (*N* = 637).

Question	Number	Percentage
1. Mrs. Sakaew shakes the bottle to mix the separated sediment with the clear liquid before taking the medicine each time.	625	98.1
2. Mrs. Noi combines different medicines into one sachet for convenience.	621	97.5
3. Ms. Lumyai takes her post-meal medication at 12:45 *p*m after finishing her meal at 12:30 *p*m	591	92.8
4. Mr. Ma stores his medicine on top of the refrigerator, which is placed next to a window.	587	92.2
5. Mrs. Bai notices that her medicine has changed colour from white to yellow and decides not to take it.	607	95.3
6. Mrs. Chan continues taking her medication despite developing a rash and does not consult a doctor.	618	97.0
7. Mr. Dang takes his medication without reading the instructions on the label.	627	98.4
8. Mr. Som immediately resumes eating after taking his pre-meal medication.	542	85.1
9. Mr. Dee, unsure about taking multiple medications simultaneously, consults a pharmacist.	610	95.8
10. Mr. Kham recognises the red label on his hospital-issued medication as indicating it is for external use only and refrains from ingesting it.	591	92.8

The MULT (Chaijinda et al., 2007) was employed to measure medicine literacy. Participants scoring 8 points or fewer were classified as having low literacy, whereas those scoring 9 points or more were deemed to have adequate drug literacy. Based on the survey, 83 (13.0%) participants were classified as having low literacy. In contrast, 554 (87.0%) participants demonstrated adequate drug literacy by scoring 9 points or higher (Table 3).

**Table 3.** The MULT score assessment among different faculty groups.

Faculty groups	Score (items)	Number	Percent
Social science (*n* = 363)	≤8	42	11.6
Average score = 9.45 ± 0.95	>8	321	88.4
Science and Technology (*n* = 136)	≤8	33	25.2
Average score = 9.23 ± 1.03	>8	103	75.8
Health sciences (*n* = 138)	≤8	8	5.8
Average score = 9.65 ± 0.73	>8	130	94.2
Overall average score (*n* = 637)	9.44 ± 0.93		

The average MULT scores across the groups were as follows: the Social sciences group scored 9.45 ± 0.95, the Science and Technology group scored 9.23 ± 1.03, and the Health sciences group scored 9.65 ± 0.73. Participants with scores greater than or equal to 9 points constituted 88.4% of the Social sciences group, 75.8% of the Science and Technology group, and 94.2% of the Health sciences group. Conversely, those scoring less than or equal to 8 points accounted for 11.6% of the Social sciences group, 25.2% of the Science and Technology group, and 5.8% of the Health sciences group (Table 3).

### USP-DI pictogram comprehension test

The average score for understanding the USP-DI pictogram was 21.82 ± 3.82 points, with scores ranging from 6 to 30 points. The distribution of scores showed that 1.0% of participants scored between 6 and 10 points, 5.0% scored between 11 and 15 points, 27.9% scored between 16 and 20 points, 48.8% scored between 21 and 25 points, and 17.5% scored between 26 and 30 points.

According to the predetermined comprehension criteria, respondents were required to achieve a score of at least 85%, equivalent to 26 points out of a total of 30 points, in alignment with the ANSI standard. The analysis revealed that 111 participants met this criterion, scoring 26 points or higher on the test, thus demonstrating their comprehension of the USP-DI pictorial drug labels. This subset represented 17.5% of the total sample.

Among the 637 respondents all participants the highest rate of correct responses pertained to pictogram 11, ‘Do not take drugs if pregnant,’ with a 99.2% accuracy rate. Conversely, the least correctly identified label was picture 7, ‘Store drugs in a refrigerator in a normal compartment,’ with only 6% accuracy. When examining the responses by group, the Social sciences group exhibited the highest accuracy for picture 11, ‘Do not take drugs if pregnant,’ with 99.7% correct responses, and the lowest accuracy for picture 7, with 0% correct responses. The Science and Technology group also had the highest accuracy for pictures 11, ‘Do not take drugs if pregnant,’ and 23, ‘Eye drops,’ both at 97.1%, whereas picture 7 again was identified with 0% accuracy. The Health sciences group showed the highest accuracy for pictures 11 and 23, with 100% correct responses for both, whereas the lowest accuracy was observed for picture 7, with 31.2% correct responses. These results are summarised in Table 4.

**Table 4.** Pictogram signs tested in this study.

No.	USP-DI Pictograms	Meaning	Faculty group (Number of samples with correct answers/Percentage)			Total (*n* = 637)
			Social science (*n* = 363)	Science and Technology (*n* = 136)	Health sciences (*n* = 138)	
1	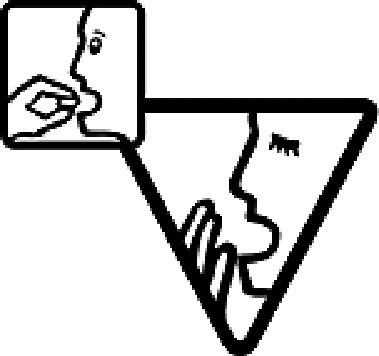	Taking this drug can cause drowsiness.	323 (89.0)	108 (79.4)	101 (73.2)	532 (83.5)
2	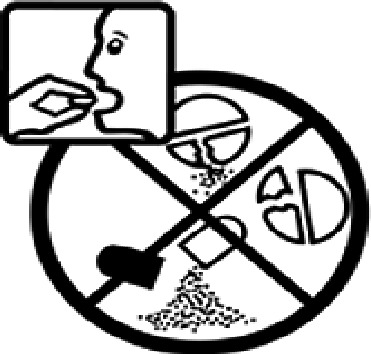	Do not break, divide, or crush tablets or unwrap capsules.	193 (53.2)	65 (47.8)	116 (84.1)	374 (58.7)
3	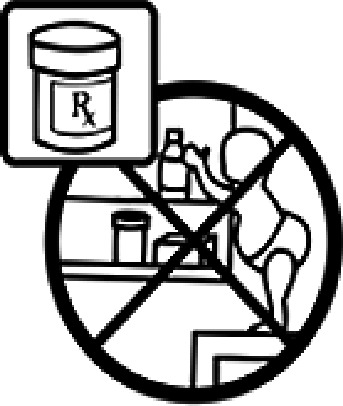	Do not keep the medicine out of the reach of children.	345 (95.0)	132 (97.1)	135 (97.8)	612 (96.0)
4	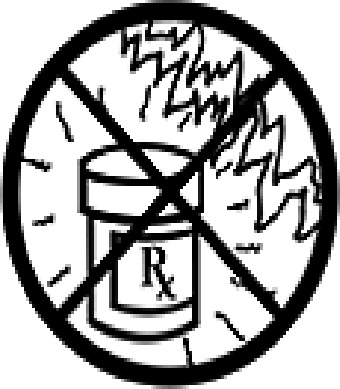	Do not store the drug near heat or sunlight.	295 (81.3)	75 (55.1)	130 (94.2)	500 (78.5)
5	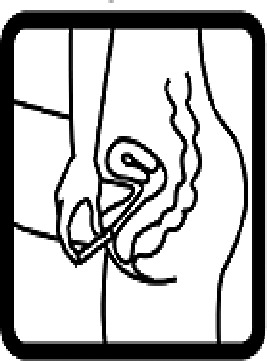	Vaginal insertion	111 (30.6)	43 (31.6)	112 (81.2)	266 (41.7)
6	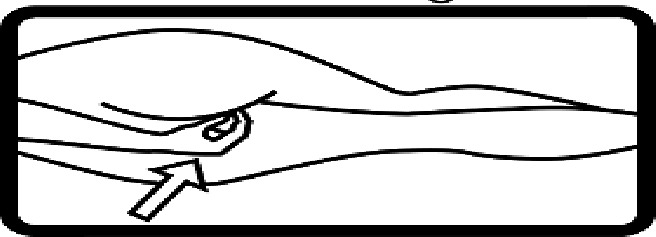	Anal suppositories	139 (38.3)	45 (33.1)	116 (84.1)	297 (46.6)
7	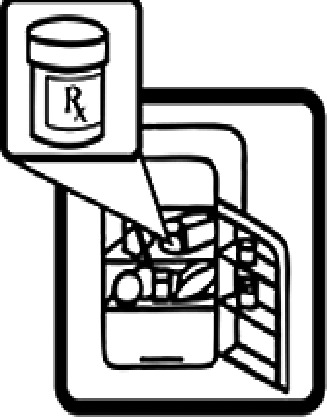	Store the drug in a refrigerator, ordinary compartment.	0 (0.0)	0 (0.0)	43 (31.2)	43 (6.75)
8	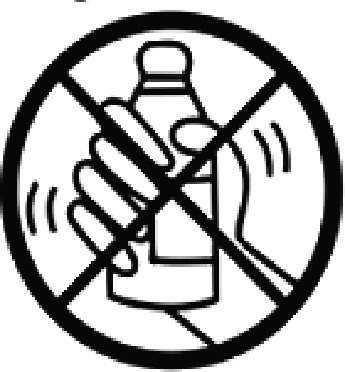	Do not shake the medicine bottle.	349 (96.1)	128 (94.1)	136 (98.6)	613 (96.2)
9	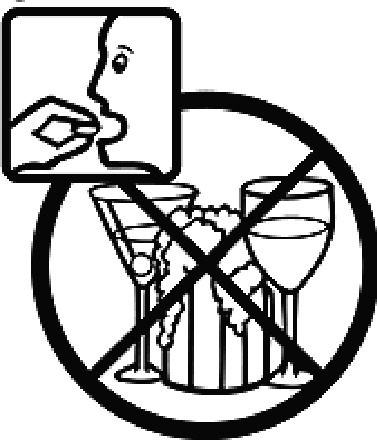	Do not drink alcohol while taking medication.	298 (82.1)	107 (78.7)	132 (95.7)	537 (84.3)
10	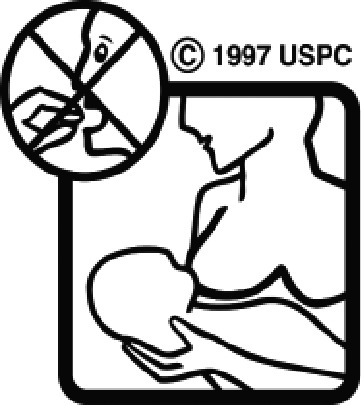	Do not take the drug in lactating women.	341 (93.9)	127 (93.4)	135 (97.8)	603 (94.6)
11	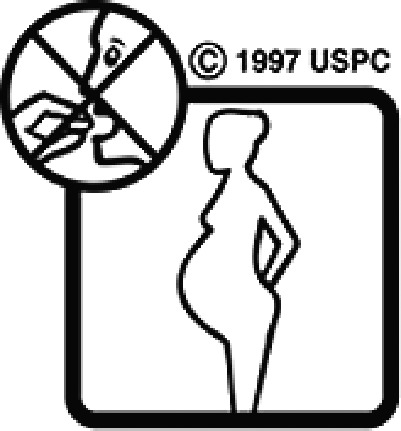	Do not take the drug in pregnant women.	362 (99.7)	132 (97.1)	138 (100)	632 (99.2)
12	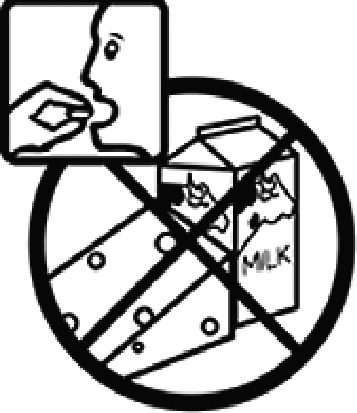	Do not take the drug with buttermilk or other dairy products.	269 (74.1)	110 (80.9)	130 (94.2)	509 (79.9)
13	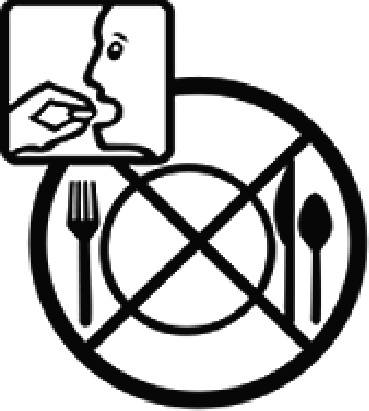	Do not take the medicine with food.	274 (75.5)	103 (75.7)	128 (92.8)	505 (79.3)
14	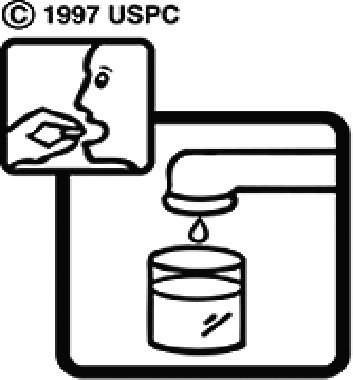	Take medicine and drink 1 glass of water.	259 (71.3)	79 (58.1)	108 (78.3)	446 (70.0)
15	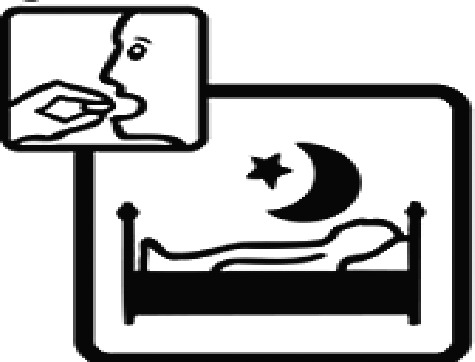	Take medication before bedtime.	333 (91.7)	119 (87.5)	137 (99.3)	589 (92.4)
16	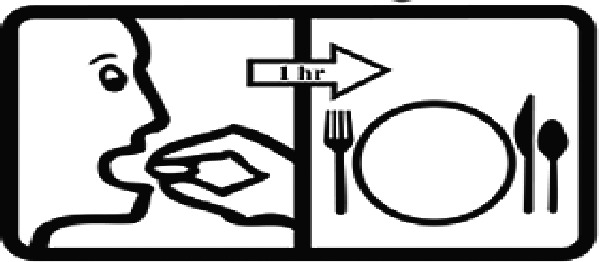	Take the drug 1 h before meals.	169 (46.6)	60 (41.1)	102 (73.9)	331 (51.9)
17	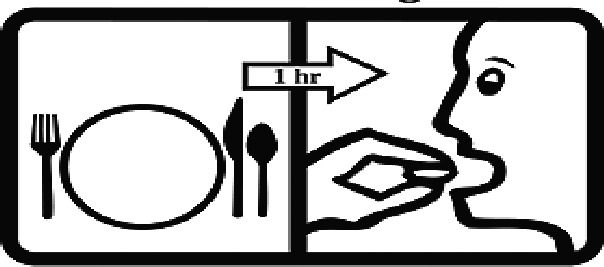	Take the medicine 1 h after meals.	163 (44.9)	61 (44.9)	104 (75.4)	328 (51.5)
18	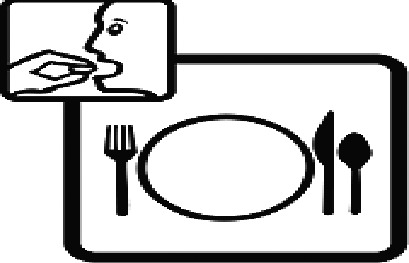	Take medicine with food.	252 (69.4)	108 (79.4)	127 (92.0)	487 (76.5)
19	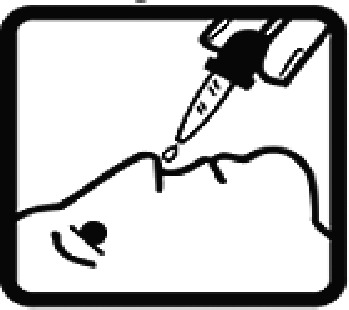	Nasal drops	309 (85.1)	118 (86.8)	136 (98.6)	563 (88.4)
20	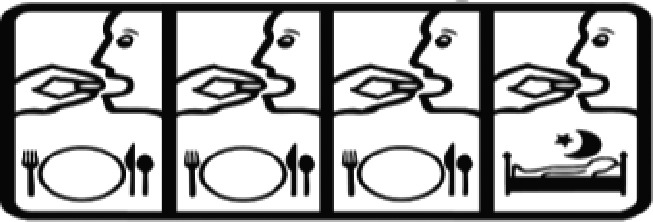	Take medicine with food in the morning, at lunchtime, in the evening, and at bedtime	23 (6.3)	6 (4.4)	37 (26.8)	66 (10.4)
21	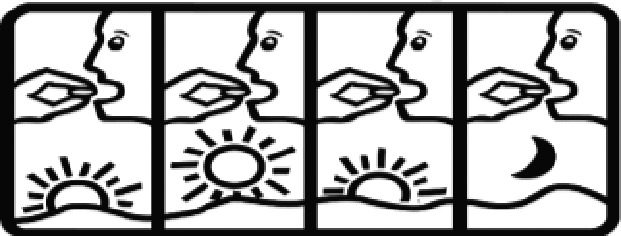	Take the medicine four times: in the morning, at lunchtime, in the evening, and before bedtime.	185 (51.0)	66 (48.5)	110 (79.7)	361 (56.6)
22	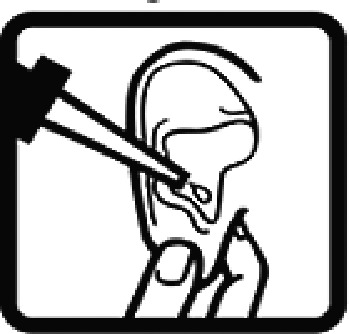	Ear drops	357 (98.3)	129 (94.9)	136 (98.6)	622 (97.6)
23	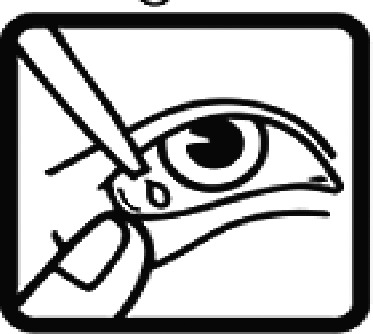	Eye drops	359 (98.9)	132 (97.1)	138 (100)	629 (98.7)
24	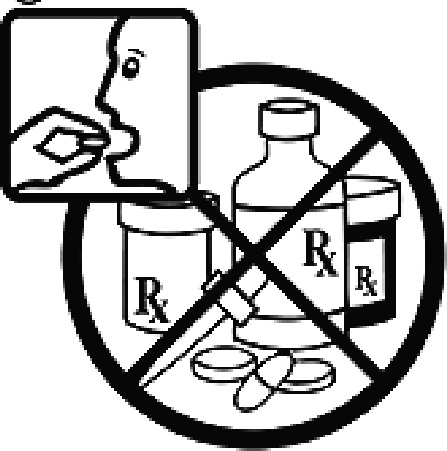	Do not take multiple medications together.	109 (30.0)	39 (28.7)	111 (80.4)	259 (40.6)
25	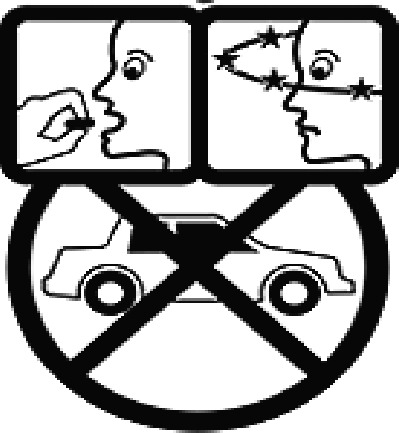	Taking the medicine causes drowsiness; do not drive.	230 (63.4)	86 (63.2)	127 (92.0)	443 (69.5)
26	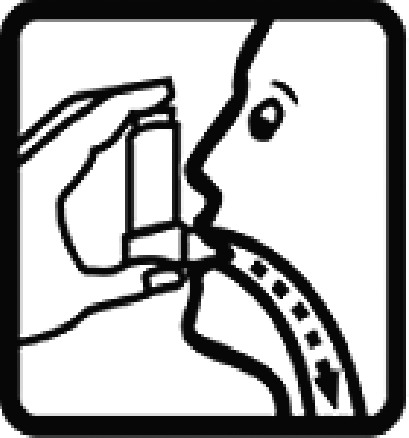	Metered dose inhaler	331 (91.2)	124 (91.2)	132 (95.7)	587 (92.1)
27	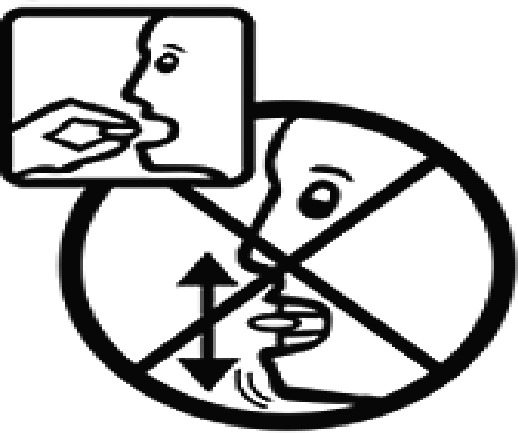	Do not chew medicine.	299 (82.4)	106 (77.9)	117 (84.8)	522 (81.9)
28	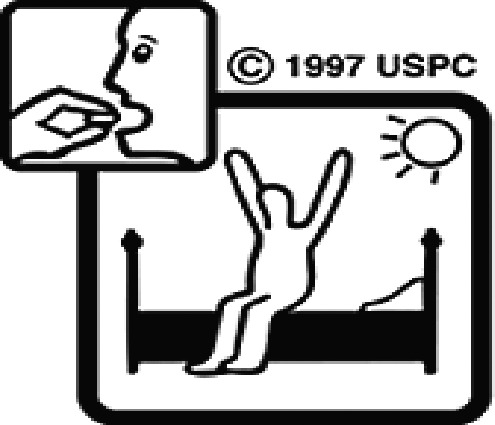	Take medicine in the morning.	271 (74.7)	106 (77.9)	136 (98.6)	513 (80.5)
29	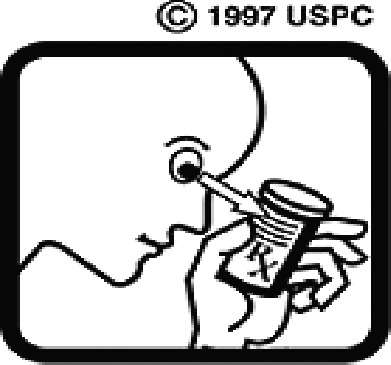	Read the label before taking the drug.	352 (97.0)	132 (97.1)	137 (99.3)	621 (97.5)
30	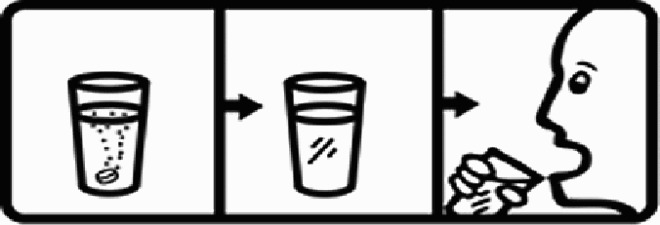	Dissolve the drug in water before taking it.	274 (75.5)	106 (77.9)	127 (92.0)	507 (79.6)

The study sample comprised 637 university students, divided into three faculty groups: Social sciences (363 students), Science and Technology (136 students), and Health sciences (138 students). Across all three faculties, the correct identification rate for pictograms was less than 50% for certain questions. In particular, questions 7, 20, and 24 had correct response rates of 6.75%, 10.4%, and 40.6%, respectively. The most frequently misinterpreted label was pictogram 5. The three most common incorrect interpretations for pictogram 5 were: (1) ‘Store the medicine in the refrigerator,’ (2) ‘Refrigerate the medicine,’ and (3) ‘Store the medicine at a cool temperature.’ For pictogram 20, the most common incorrect interpretations were: (1) ‘Take the medicine as prescribed by the doctor,’ (2) ‘Take the medicine at the specified time,’ and (3) ‘Take the medicine four times daily morning, noon, evening, and before bedtime.’ For pictogram 24, the most common incorrect interpretations were: 1) ‘Dangerous drugs,’ (2) ‘Medicines without drug labels,’ and (3) ‘External drugs,’ as shown in Table 7. The MULT and USP-DI pictogram comprehension revealed that the Health sciences group demonstrated the highest proficiency, followed by the Social sciences and Science and Technology groups, with statistically significant differences (*P* < 0.05), as shown in Table 5.

**Table 5.** Comparison of total scores between groups.

Score	Faculty Groups	Mean ± SD	*P*-value
Overall score for drug literacy	Social sciences vs. Science and Technology	9.45 ± 0.94 vs. 9.23 ± 1.03	0.02
	Social sciences vs. Health sciences	9.45 ± 0.94 vs. 9.65 ± 0.73	0.03
	Science and Technology vs. Health sciences	9.23 ± 1.03 vs. 9.65 ± 0.73	0.00
Overall score for comprehension of USP-DI pictograms	Social sciences vs. Science and Technology	20.86 ± 3.26 vs. 20.23 ± 3.53	0.04
	Social sciences vs. Health sciences	20.86 ± 3.26 vs. 25.89 ± 2.36	0.00
	Science and Technology vs. Health sciences	20.23 ± 3.53 vs. 25.89 ± 2.36	0.00

Based on the evaluation encompassing the MULT scores and USP-DI pictograms, significant differences were observed. Highly literate individuals scored an average of 9.74 ± 0.43 in MULT, whereas those with lower literacy scored 7.48 ± 0.99, demonstrating statistical significance. Similarly, in the assessment of the comprehension of USP-DI pictogram, highly literate participants averaged 22.04 ± 3.79 points, compared to 20.32 ± 3.66 points among less literate individuals, also showing statistical significance (*P* < 0.001), as detailed in Table 6.

**Table 6.** Comparison of the overall MULT score and comprehensive USP-DI Pictograms score.

Score	Low literate skills	High literate skills	*P*-value
Overall MULT score	7.48 ± 0.99	9.74 ± 0.43	0.00
Overall score for comprehension of USP-DI pictograms	20.32 ± 3.66	22.04 ± 3.79	0.00

**Remark**; MULT: Medication Use Literacy Test.

### The relationship between factors and the comprehensive of USP-DI pictograms

The univariate analysis revealed significant correlations between the level of comprehension of the USP-DI pictograms and factors such as age, average educational grade, and faculty group (Table 7). Further logistic regression analysis indicated that these factors were significantly associated with comprehension levels of the USP-DI pictogram. Age, average educational grade, and faculty group emerged as influential variables. The analysis demonstrated that individuals over the age of 20 exhibited a lower level of understanding of USP-DI pictogram compared to those under 20 years old (OR = 0.47; 95% CI: 0.25–0.88; *P* = 0.02), Cumulative GPA <3.00 (OR = 0.55; 95%CI: 0.32–0.96; *P* = 0.04) and other faculties (OR = 0.04; 95%CI: 0.02–0.08; *P* < 0.001).

**Table 7.** Factors affecting the understanding of drug labels.

Factor	Number (percentage)	Level of understanding of USP-DI pictograms		Adjusted OR (95%CI)	*P*-value
		<85%	≥85%		
Gender					
Male	178 (27.9)	152 (28.9)	26 (23.4)	1	0.24
Female	459 (72.1)	374 (71.1)	85 (76.6)	0.24 (0.37–1.28)	
Age (years)					
<20	355 (55.7)	333 (63.3)	22 (19.8)	1	0.02*
≥20	282 (44.3)	193 (36.7)	89 (80.2)	0.47 (0.25–0.88)	
History of hospitalisation					
Less than 1 time/month	409 (64.2)	333 (63.3)	76 (68.5)	1	0.65
More than 1 time/month	228 (35.8)	193 (36.7)	35 (31.5)	0.65 (0.36–1.18)	
Comprehension rating on how to take the drug					
Comprehension of how to take the drug ≥8 points	554 (87.0)	447 (85.0)	107 (96.4)	1	0.11
Comprehension of how to take the drug <8 points	83 (13.0)	79 (15.0)	4 (3.6)	0.38 (0.12–1.24)	
GPAX					
3.00–4.00	284 (44.6)	210 (39.9)	37 (33.3)	1	0.04*
<3.00	353 (55.4)	316 (60.1)	74 (66.7)	0.55 (0.32–0.96)	
Faculty groups					
Faculty of Health sciences group	138 (21.7)	51 (9.7)	87 (78.4)	1	<0.001*
Other faculties	499 (78.3)	475 (90.3)	24 (21.6)	0.04 (0.02–0.08)	

**Remark**; GPAX: Cumulative Grade Point Average.

## Discussion

In this survey on the comprehension of USP-DI pictorial drug labels among university students, the majority of respondents were female (72.1%). This gender distribution aligns with the participant demographics, where females aged ≤20 years represent 55.7%. The Faculty of Accounting and Management had the highest number of respondents (22.0%), whereas the Faculty of Cultural Sciences had the lowest. This distribution corresponds to varying student populations across different faculties at the university. The highest GPA range among respondents was 3.00–3.49, accounting for 21.2%. Additionally, most respondents (90.4%) reported no underlying diseases, and the most common family size (52.4%) was four members. The frequency of hospital visits was predominantly less than once per month (52.6%), and the average monthly income was in the range of 5001–10,000 baht (25.7%).

Findings from the MULT assessment indicate that the majority of undergraduate respondents demonstrated adequate medication-use literacy, with 88.6% classified as having high literacy levels. Notably, item-level analysis revealed that while most students correctly identified obvious medication misuse behaviours (e.g. 98.4% correctly responded that ‘Mr. Dang took the medicine without reading the label’), comprehension decreased when interpretation of administration timing (‘Mr. Som received pre-meal medicine,’ 85.1%) or situational judgment (‘For the convenience of carrying medicines,’ 35.8%) was required. These discrepancies suggest that, although general awareness of safe medication practices is relatively strong, students may struggle with more nuanced or context-based scenarios. This observation is consistent with a previous study reporting substantially lower adherence and comprehension rates among individuals with limited formal education in Northeastern Thailand, emphasising the impact of literacy and contextual understanding on medication-use behaviours (Phimarn et al., 2019).

Further analysis by academic discipline revealed a statistically significant difference in MULT scores, with students from Health Sciences faculties achieving the highest average score (9.65 ± 0.73), followed by those in Social Sciences and Science and Technology. This gradient may be attributed to curriculum exposure, prior training in drug-related topics, or increased health-related awareness, as similarly suggested in prior studies on HL determinants (Nutbeam, 2008; Pouliot et al., 2018). The higher proportion of high-literacy individuals in health-related faculties (94.2%) also aligns with the literature, which indicates that domain-specific knowledge – especially regarding pharmacotherapy – enhances comprehension of medication-related content (Zhang et al., 2014).

These findings highlight the importance of incorporating structured medication literacy education across all disciplines, not only within pharmacy or health science programmes. Given the role of university students in informal caregiving and health communication within families – especially in multigenerational Thai households – this gap highlights a public health opportunity for targeted literacy interventions beyond health faculties.

The overall comprehension of USP-DI pictograms among participants was below the internationally recommended benchmark for acceptable understanding (ANSI, 2011). This raises important concerns regarding the effectiveness of standardised pictograms when applied in the Thai undergraduate context, particularly among students outside the health sciences. The limited attainment of the recommended comprehension threshold suggests that USP-DI pictograms, originally developed in Western contexts, may not be universally interpretable across diverse cultural and educational backgrounds. These results highlight the potential need for localised adaptation and targeted educational efforts to improve the interpretability and utility of pharmaceutical pictograms in multicultural settings.

Our findings highlight the limitations of applying internationally developed pictograms without cultural adaptation. Although consistent with the study by Dowse and Ehlers, which demonstrated the effectiveness of locally adapted pictograms for low-literacy patients in South Africa, our results emphasise the importance of cultural contextualisation (Dowse & Ehlers, 2005). In contrast, another study reported that 61% of Indian non-pharmacy students could accurately interpret USP-DI pictograms and preferred them to traditional labels, suggesting that comprehension levels may vary significantly depending on population exposure, visual familiarity, and educational background (Mishra et al., 2011).

The lower performance observed in our study may reflect limited formal exposure to pictogram-based labelling, especially outside pharmacy curricula, and emphasises the need to incorporate pictogram education into broader health communication efforts. Moreover, these findings raise questions about the universal applicability of the ANSI 85% threshold in diverse sociocultural contexts. It may be necessary to either recalibrate comprehension expectations or prioritise the development of culturally relevant pictograms co-designed with local users to ensure usability and interpretability (Chaijinda et al., 2007; Katz et al., 2006).

The marked variation in USP-DI pictogram comprehension across academic disciplines suggests that domain-specific education plays a critical role in shaping students’ ability to interpret visual health communication tools. Students in health sciences consistently demonstrated stronger comprehension, likely as a result of their greater exposure to pharmacological concepts, medical terminology, and patient-centered communication as part of their academic training. In contrast, students from non-health disciplines may lack the foundational understanding necessary to accurately interpret medication-related pictograms, highlighting the influence of curricular content on visual literacy in healthcare contexts. These findings support the need to integrate medication-related visual literacy into broader educational strategies to enhance comprehension among diverse student populations.

Our findings align with previous research indicating that students enrolled in health-related programmes tend to exhibit higher levels of pictogram comprehension as a result of greater curricular exposure to pharmaceutical labelling and patient counselling materials (Banstola, 2013; Dowse, 2023). Notably, Banstola (2013) found that even first-year pharmacy students in India demonstrated strong understanding of USP-DI pictograms, suggesting that early and repeated engagement with such materials can significantly enhance visual health communication competencies (Banstola, 2013). This supports the broader assertion that structured educational interventions play a critical role in developing functional medication literacy.

However, a key difference in our study is the broader inclusion of students from non-health disciplines, which revealed pronounced disparities in comprehension levels. Unlike Banstola’s pharmacy-focused cohort, our study captured variability across diverse academic backgrounds. This highlights a potential gap in general education curricula, where students not formally trained in health sciences may lack the necessary exposure to accurately interpret pharmaceutical pictograms.

These findings emphasise the importance of contextual adaptation and interdisciplinary HL efforts, particularly in multicultural settings like Thailand, where students often support family members in medication management. Our study contributes novel evidence by demonstrating that even within a shared cultural setting, academic discipline significantly influences pictogram comprehension an insight that may inform the development of targeted visual communication strategies in public health and pharmacy education.

However, our findings must be interpreted considering contextual limitations. Although some studies have reported high pictogram comprehension when symbols are culturally adapted (Chaijinda et al., 2007), our results reflect the challenges of using standardised, non-localised USP-DI pictograms in a Thai university setting. Indeed, prior research from African and Southeast Asian contexts has highlighted the need for cultural tailoring of visual aids to maximise comprehension across diverse populations (Dowse & Ehlers, 2001; Katz et al., 2006).

Furthermore, qualitative feedback reported by Dowse et al. (2023) offers additional insight, showing that pharmacy students view pictograms as valuable tools for bridging language barriers, especially among low-literacy patients. However, they also emphasised the importance of improving pictogram clarity and realism points echoed by some of our respondents. These observations highlight the need not only to enhance pictogram design but also to incorporate pictogram training into undergraduate curricula beyond health sciences to improve public understanding of medication information (Dowse, 2023).

This study identified several key factors significantly associated with higher pictogram comprehension: being over the age of 20, having a cumulative GPA greater than 3.00, and enrolment in a Health Sciences faculty. These findings suggest that cognitive maturity and academic performance may play critical roles in accurately interpreting medication pictograms. Older students may benefit from greater life experience or prior exposure to health-related materials, enhancing their ability to contextualise visual symbols. Similarly, students with higher academic achievement may possess stronger cognitive and analytical skills that facilitate interpretation of abstract or symbolic information (Kassam et al., 2010; Katz et al., 2006).

The significantly better performance among health sciences students is likely attributable to formal exposure to pharmaceutical content, including labelling and patient communication, as part of their curriculum. This is consistent with findings from previous studies that reported higher pictogram comprehension among pharmacy students and emphasised the role of structured health education in developing visual literacy related to medication use (Banstola, 2013; Dowse, 2023). It is also possible that prior instruction or clinical training enabled these students to more accurately infer or ‘guess’ the meaning of unfamiliar pictograms through deductive reasoning.

However, it is important to note that reliance on prior comprehension and academic background may disadvantage students from non-health disciplines, potentially contributing to health disparities in medication comprehension. This highlights the need for interdisciplinary integration of basic medication literacy and pictogram interpretation into general education curricula, particularly for university students who often serve as informal health advisors within their households in low- and middle-income settings like Thailand.

The current study found that participants with higher medication-use literacy, as measured by the MULT, demonstrated significantly better comprehension of USP-DI pictograms. This suggests that functional HL may play a critical role in the accurate interpretation of visual medication instructions. These findings are consistent with a previous study that reported demographic and cognitive factors particularly age, education level, and occupation were stronger predictors of pictogram guessability than routine behaviours such as reading drug labels or purchasing medications. That study also emphasised that perceptions of pictogram clarity and cognitive load influenced interpretive accuracy (Chan & Chan, 2013).

The association between high literacy levels and superior pictogram comprehension observed in our study likely reflects underlying cognitive advantages, such as better visual processing, information recall, and health-related schema development. This aligns with prior studies showing that individuals with limited literacy skills face challenges in interpreting pictograms, which may compromise safe medication use (Braich et al., 2011; Mwingira & Dowse, 2007). Kassam et al. (2010) further demonstrated that pictogram comprehension is directly related to baseline literacy, highlighting the importance of tailoring pictographic communication to the cognitive capacities of target populations (Kassam et al., 2010).

Additionally, our findings reinforce broader evidence indicating that higher educational attainment correlates with greater accuracy in interpreting drug-related visual symbols (Banstola, 2013). However, this contrasts with a previous study that observed comparable levels of pictogram comprehension between pharmacy and non-pharmacy students in Pakistan (Yasmin et al., 2014). The discrepancy may be attributed to contextual factors such as the specific pictograms used, cultural familiarity with the symbols, or differences in participant selection. These divergent results highlight the importance of validating pictograms within the local sociocultural and educational context before implementing them in public health communication.

Collectively, these findings point to the need for improved pictogram design and targeted HL education, especially for populations with limited exposure to medication information. Pictograms should be tested not only for graphic clarity but also for cognitive accessibility, particularly when deployed in diverse educational settings.

This study indicates that the implementation of the USP-DI’s international pictorial drug labels among Thai individuals still faces challenges, particularly in interpreting specialised drug labels. However, this study acknowledges the limitations of being a single-population study confined to a specific regional or cultural context. Future studies should explore diverse populations to elucidate these outcomes.

Pictograms not only aid pharmacy professionals in patient communication but also reinforce verbal information and prompt patients to recall information effectively, an aspect noted by several students. Nevertheless, the anecdotal feedback suggests that some students may lack awareness of these roles, indicating the need for training on the rationale and effective use of pictograms. Most students viewed the current pictogram designs as high quality, relevant, clear, culturally appropriate, and effective for conveying medication-related information. Suggestions for improvement include modifying underperforming pictograms to enhance clarity, with some advocating for the inclusion of more explanatory text. This suggestion aligns with the findings of a recent study wherein improvements in pictogram comprehension through graphical and textual adjustments, on enhancing comprehension of pictogram, were demonstrated (Dowse et al., 2023).

The strengths of this study are as follows. First, the notable aspects of this study include the high response rate and the significant level of student involvement in the survey. All Likert scale items were fully answered, and intelligent, insightful, and valuable responses to the open-ended questions were particularly noteworthy. Second, data collection was facilitated through self-administered questionnaires, which encouraged respondents to freely disclose information, particularly on matters of comprehension that might not have been openly shared in the interviews. This methodological approach enhances the reliability of the academic outcomes. Third, the findings provide a valuable foundation for enhancing comprehension about medical use among students, offering opportunities to improve educational interventions and providing targeted information to enhance understanding of this critical area.

### Limitations

This study had some limitations. First, the tracking of data collection was challenging because of student internships, particularly among 5th and 6th year students at the Faculty of Medicine, which may have impacted the response rates and consistency in data collection. Second, although we utilised USP-DI picture labels, they may not have encompassed all the commonly encountered daily medication recommendations, potentially limiting the generalizability of the findings. Third, the predominance of female participants in the sample from only one university may not fully represent gender diversity across institutions, influencing the broader applicability of the study outcomes. Furthermore, the underrepresentation of students from smaller faculties (e.g. only 1–2 participants) may limit generalizability to the entire university population. We recommend that future studies employ stratified or quota sampling to ensure proportionate representation across faculties. Nonetheless, this study provides valuable preliminary insights into students’ comprehension of medication pictograms across a diverse academic cohort.

Future research should explore the factors influencing drug literacy and comprehension of pictorial drug labels. This would provide broad insights into these critical areas. Additionally, the development of drug labels that are culturally and contextually relevant should be emphasised and implemented in university pharmacies. This approach should facilitate the use of medications requiring specific techniques, thereby enhancing the usability and accuracy of medication administration.

## Conclusion

This study reveals substantial differences in the comprehension of USP-DI pictograms among Thai undergraduate students, with those in health sciences programmes demonstrating significantly higher proficiency. Although overall medication-use literacy was adequate, many students particularly those from non-health disciplines or with lower academic performance struggled to interpret standardised pictograms. These findings highlight the importance of incorporating pictogram education into broader HL initiatives, extending beyond pharmacy curricula to include all university students. This is particularly relevant in contexts where students often support family members in medication management. In multicultural and multilingual settings, culturally appropriate and contextually adapted pictograms can enhance patient understanding, especially among individuals with low literacy or limited language proficiency. For pharmacy practice, the results support the use of visual aids in patient counselling to bridge communication gaps. Ultimately, the study emphasises the need for a more inclusive, cross-disciplinary approach to health education and patient communication strategies that prioritise accessibility, clarity, and cultural relevance.

## Supplementary Material

Certificate of editing.pdf

## Data Availability

Data supporting the findings of this study are available upon request from the corresponding author. The data are not publicly available due to ethical restrictions.
